# Usefulness of Nutritional Intervention Through New Digital Technologies in Patients with Inflammatory Bowel Disease

**DOI:** 10.3390/nu18060910

**Published:** 2026-03-13

**Authors:** Cristina Suárez Ferrer, I. Martorell Mariné, J. L. Rueda García, C. Cubillo García, L. García Ramírez, C. Amiama Roig, M. Sánchez Azofra, J. Poza Cordon, E. Martin Arranz, C. García-Rojas Pleite, J. Noci Belda, Maria Dolores Martin-Arranz

**Affiliations:** 1 Gastroenterology Department, Health Research Institute (IdiPAZ), La Paz University Hospital, Paseo de la Castellana 261, 28046 Madrid, Spain; ruedagarcia.joseluis@gmail.com (J.L.R.G.); eiiclapaz.enfermeria@gmail.com (C.C.G.); eiiclapaz@gmail.com (L.G.R.); camiamaroig2@gmail.com (C.A.R.); maryazofra@gmail.com (M.S.A.); joaquin.poza@salud.madrid.org (J.P.C.); edumartinarranz@gmail.com (E.M.A.); eic.lapazcgp@gmail.com (C.G.-R.P.); mmartinarranz@salud.madrid.org (M.D.M.-A.); 2 Nutrition Department, Nootric Inc., 08940 Barcelona, Spain; imartorell@nootric.com; 3 Gastroenterology Department, La Paz University Hospital, 28046 Madrid, Spain; jesusa.noci@salud.madrid.org

**Keywords:** inflammatory bowel disease, nutrition, mobile health, Mediterranean diet, malnutrition screening, diet quality

## Abstract

**Background:** Malnutrition and suboptimal diet quality are common, yet under-recognized, in inflammatory bowel disease (IBD) and are associated with worse clinical outcomes and lower quality of life. Digital tools may facilitate continuous, personalized nutritional support, but evidence in IBD remains limited. The aim of this study was to evaluate the impact of a nutritional intervention based on a mobile application (Nootric^®^) on nutritional status, diet quality, and malnutrition risk in patients with IBD undergoing stable follow-up. **Methods:** We conducted a prospective longitudinal cohort study without a control group including 151 adult patients with Crohn’s disease or ulcerative colitis under stable follow-up in a tertiary IBD unit. Participants used a structured digital nutritional support program through the Nootric^®^ app for 24 weeks, supervised by dietitians and the IBD team. Clinical activity, biochemical markers (C-reactive protein, fecal calprotectin), nutritional biomarkers (albumin, prealbumin, micronutrients), body mass index (BMI), malnutrition risk (self-administered Malnutrition Universal Screening Tool, MUST), and diet quality (PREDIMED and an expanded “Nootric score”) were assessed at baseline, 12 weeks, and 24 weeks. Analyses focused on patients with adequate adherence. **Results:** Of the 151 included IBD patients, 110 maintained stable app use. Mean albumin increased from 4.38 to 4.49 g/dL at 24 weeks (*p* = 0.003), and prealbumin from 24.9 to 26.1 mg/dL (*p* = 0.047), despite the absence of overt protein–calorie malnutrition at baseline. Patients with obesity achieved a mean weight loss of approximately 6% of baseline body weight. Diet quality improved significantly, with higher Nootric score and a positive correlation between app use intensity and increased score. Malnutrition risk according to the MUST scale improved in more adherent patients, while clinical and biochemical disease activity remained stable overall. **Conclusions:** A mobile app-based nutritional program supervised by dietitians was feasible, well accepted, and associated with improved nutritional markers, diet quality, and malnutrition risk, supporting its role as a complementary component of IBD care.

## 1. Introduction

Nutrition plays a crucial role in the management and treatment of inflammatory bowel disease (IBD).

Inflammatory bowel disease (IBD) comprises two main entities: Crohn’s disease (CD), which can affect any segment of the gastrointestinal tract and is characterized by transmural inflammation, and ulcerative colitis (UC), which is limited to the colonic mucosa and presents with continuous inflammation starting in the rectum. Both conditions are chronic, immune-mediated disorders with a relapsing–remitting course and may require long-term immunomodulatory or biologic therapies. Patients with IBD often face nutritional challenges due to chronic intestinal inflammation, which can impair nutrient absorption, increase metabolic requirements, reduce appetite, and cause symptoms such as diarrhea and abdominal pain; these factors may lead to weight loss, micronutrient deficiencies, and sarcopenia, which in turn are associated with poorer clinical outcomes and reduced quality of life [1,2].

Traditionally, malnutrition in IBD has been linked to protein–calorie undernutrition; however, it is now recognized that it can also occur in individuals with normal weight or even overweight and obesity [3]. This highlights that an apparently adequate caloric intake does not always guarantee an optimal nutritional status, as many patients present unbalanced diets and deficiencies of essential micronutrients. Vitamin and mineral deficiencies, together with alterations in body composition, are common in IBD and can exacerbate intestinal inflammation, compromise immune response, and hinder mucosal healing [4]. Therefore, maintaining an adequate nutritional status is a fundamental component of comprehensive IBD management. In this context, nutritional support should be considered a key therapeutic tool, which must be individually tailored to the needs and characteristics of each patient [5].

In the existing literature, the use of restrictive diets or the indiscriminate elimination of food groups in these patients is currently discouraged [6,7]. These practices, often adopted empirically by patients themselves, can lead to significant nutritional deficiencies and further worsen malnutrition. In contrast, current evidence suggests that following a dietary pattern based on the Mediterranean diet may be associated with better control of inflammation, a more balanced gut microbiota, and improved overall nutritional status [8]. The Mediterranean diet is characterized by a high intake of fruits, vegetables, legumes, whole grains, nuts, olive oil as the principal source of fat, moderate consumption of fish and dairy products, and low intake of red meat and ultra-processed foods.

The European Society for Clinical Nutrition and Metabolism (ESPEN) [8,9] recommends an individualized nutritional approach in IBD, tailored disease phase, extent of intestinal involvement, and patient clinical characteristics. This approach includes both the prevention and treatment of malnutrition, correction of specific micronutrient deficiencies, and dietary counseling aimed at promoting balanced eating patterns. They also emphasize that nutritional support, when indicated, should be integrated with medical treatment rather than replacing it, with multidisciplinary collaboration among gastroenterologists, dietitians–nutritionists, and other healthcare professionals being a key element in the overall management of IBD.

However, there is currently no standardized nutritional support in IBD units in our setting, despite frequent demand from healthcare professionals and patients. Furthermore, active screening for malnutrition risk is uncommon in clinical practice, leading to frequent underdiagnosis [10,11].

In summary, the inclusion of nutritional support by dietitians–nutritionists in IBD clinics could significantly enhance care quality and comprehensive disease management. Digital nutritional support tools are emerging as a promising strategy to improve dietary monitoring, nutritional education, and communication between patients and healthcare professionals. However, evidence regarding their specific effectiveness in patients with IBD is still limited. Therefore, the present study was designed to evaluate the impact of a digital nutritional support tool (Nootric^®^) in IBD patients, assessing its usefulness as a complement to standard care and its potential contribution to improving the nutritional approach in this population.

## 2. Materials and Methods

### 2.1. Study Design

This was a prospective longitudinal cohort study without a control group conducted between June 2024 and September 2025 in a tertiary IBD unit (Hospital Universitario La Paz, Madrid, Spain).

The study objectives were to assess a possible improvement in malnutrition risk parameters after the use of the digital tool (Nootric^®^), including both score-based assessments and analytical nutritional parameters. Nootric^®^ (https://www.nootric.com/) is a commercially available digital nutritional platform that enables structured dietary plans, educational modules, and direct communication with certified dietitians. In this study, its use was supervised by both dietitians and the IBD medical team.

Additionally, the study aimed to evaluate whether, in patients without malnutrition risk, this tool improves adherence to the Mediterranean diet (according to dietary recommendations established for IBD).

### 2.2. Participants

Adult patients (≥18 years) with an established diagnosis of Crohn’s disease or ulcerative colitis were consecutively recruited during routine outpatient visits. Inclusion required clinical stability and ability to use the digital platform. Exclusion criteria included severe flare, recent surgery (<3 months), pregnancy, or inability to use the application.

### 2.3. Digital Nutritional Intervention

All included patients underwent baseline, 12-week and 24-week visits following initiation of dietary recommendations through the mobile digital tool. At these visits, the MUST index, weight and height, quality of life (IBDQ-32), and laboratory tests with a complete nutritional profile were assessed. Clinical and biochemical activity of IBD was also evaluated. In addition to scheduled visits, all patients had continuous follow-up through the Nootric^®^ digital support service, as well as clinical follow-up as indicated by routine clinical practice in their IBD unit.

Participants were registered on the Nootric^®^ digital platform using an identifier code provided by the professional. Patients completed initial forms aimed both at data collection and at guiding the dietitian–nutritionist in developing a personalized dietary plan. The digital nutritional intervention was structured into weekly modules with educational content and interactive tools (challenges, dietary guides, and direct interaction with the dietitian–nutritionist through the application’s chat). The recommendations provided by Nootric^®^ were developed by qualified personnel and supervised by the IBD Unit medical team.

The program did not replace medical treatment at any time nor aim to modify IBD inflammatory activity through dietary advice alone. In the event of symptoms compatible with disease activity or complications, patients were instructed to contact the IBD unit through the usual channels. Dietary recommendations were always aligned with national and international clinical practice guidelines in gastroenterology and nutrition.

Adequate adherence was predefined as use of the application on ≥50% of days during follow-up, based on digital health engagement standards. Per protocol analyses was performed.

### 2.4. Outcomes and Assessments

Malnutrition risk was evaluated using the validated MUST tool (Malnutrition Universal Screening Tool) [12], and anthropometric measurements (weight and height) were recorded at each visit.

Clinical activity was measured using the Harvey–Bradshaw Index (HBI, <5 points to define clinical remission in CD) and the Walmsley Index (SCCAI, <2 points for UC). Laboratory monitoring included biochemical markers (fecal calprotectin (FC) < 150 µg/g and C-reactive protein (CRP) < 5 mg/dL to define biochemical remission), nutritional biomarkers (albumin, prealbumin, total proteins, cholesterol) and micronutrients (iron profile, magnesium, vitamins A, D, E, B12, and folic acid). Nutritional deficiencies were defined as any value below the lower limit of our site’s reference laboratory range.

Quality of life was assessed using the IBDQ-32 questionnaire, which includes four domains (intestinal, systemic, social, and emotional) and is widely used and validated in patients with IBD.

Baseline patient data were also recorded, such as sex, age, toxic habits, comorbidities (hypertension, dyslipidemia, diabetes mellitus, heart disease, or chronic liver disease), date of diagnosis, previous and current treatments, and IBD-related surgical history.

Before starting the program, the PREDIMED questionnaire [13,14] (PREvention with MEDiterranean Diet) was administered to evaluate adherence to the Mediterranean diet. This validated questionnaire consists of 14 questions, and low scores have been associated with increased cardiovascular risk. Additional desirable variables related to hygienic–dietary habits for patients with IBD, such as physical activity and smoking habits, were also included. Overall, this scale was termed the Nootric score, with a maximum score of 58 points for IBD. The Nootric score were reassessed at 12 and 24 weeks.

The study did not aim to evaluate pharmacological or therapeutic treatments; therefore, diagnostic and treatment guidelines established in routine clinical practice were followed throughout.

### 2.5. Statistical Analysis

A descriptive analysis of baseline cohort characteristics and of clinical, nutritional, and analytical variables collected during follow-up was performed. Continuous variables were expressed as mean and standard deviation (SD) or median and interquartile range (IQR), depending on their distribution, while categorical variables were described using absolute frequencies and percentages. Normality of continuous variables was assessed using the Kolmogorov–Smirnov test. For comparison of continuous variables between two time points, paired Student’s *t*-test or Wilcoxon test was used, as appropriate. For comparisons among more than two time points (baseline, 12 and 24 weeks), non-parametric tests for paired data (Friedman test) or Kruskal–Wallis test were used, depending on the nature of the variable analyzed. Categorical variables were compared using the χ^2^ test or Fisher’s exact test, when necessary.

Specific subgroup analyses were performed according to body mass index (BMI) (normal weight, overweight, and obesity), evaluating differences in clinical, biochemical, and nutritional parameters. The association between application use and improvement in the Nootric score was analyzed using Spearman correlation.

Lifestyle and dietary habits were assessed using the 14-item PREDIMED questionnaire and additional structured questions incorporated into the Nootric score, including physical activity frequency, fruit and vegetable intake, red meat consumption, and intake of ultra-processed foods. Changes over time were analyzed using repeated-measures statistical tests. Correlations between application use intensity and improvement in diet quality scores were assessed using Spearman correlation analysis.

Baseline Nootric score values were also compared between adherent and non-adherent patients using the Mann–Whitney U test. Final analyses were conducted only in patients with adequate adherence to the digital nutritional program. Statistical significance was set at *p* < 0.05. All statistical analyses were performed using Stata version 16 for Mac.

Prior to study initiation, a sample size calculation was performed assuming a malnutrition prevalence of 13%, a 5% error, and a 95% confidence interval. Considering a total population of 2000 patients under follow-up in the IBD unit, the required sample size was estimated to be approximately 150 patients.

### 2.6. Ethical Considerations

The study was approved by the Ethics Committee of Hospital Universitario La Paz prior to its initiation, with favorable approval on 1 July 2024 (code PI-6741). The study was conducted in accordance with the Declaration of Helsinki. All patients signed the informed consent form before participating in the study.

#### Data Protection

To access the Nootric^®^ application, participants were required to register using their email, password, name, gender, age, weight, and height. User passwords were stored encrypted in the database and rendered unreadable.

Data were stored pseudonymized to prevent linking health data to patients’ personal information. The database was encrypted, and the system keeps a record of all access, modification, and deletion of patient data by healthcare personnel.

Nootric^®^ shared data with parties involved in this study, and data will not be used for any purpose other than conducting this research and clinical practice. Data will only be published in scientific articles.

All communications between user and server, or between user and nutritionist, were encrypted using SSL/TLS. In addition, connections between devices and servers were made through an API requiring authentication and authorization for data access.

## 3. Results

**Figure 1:**
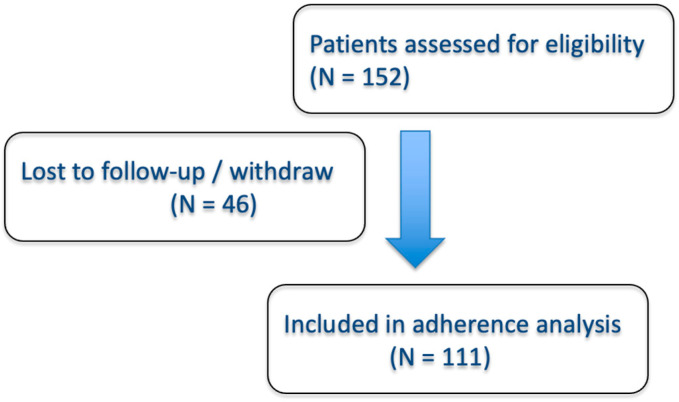
Study Flowchart.

A total of 151 patients with an established diagnosis of IBD were included (40.9% CD and 59.1% UC). Among them, 110 patients had stable follow-up of nutritional recommendations through the mobile application. The flowchart summarizing the inclusion and follow-up of patients is presented in Figure 1. In our sample, 53% were women and the mean age was 46.8 years (SD: 12.8). Other baseline characteristics and IBD-related data are summarized in Table 1.

Regarding the nutritional status of included patients from an analytical perspective, no patients met criteria for protein undernutrition or significant micronutrient deficiencies on average in the cohort prior to the nutritional intervention.

However, mean albumin levels (g/dL) increased significantly from a baseline value of 4.38 (SD: 0.26) to 4.45 (SD: 0.28) at 12 weeks and to 4.49 (SD: 0.26) at 24 weeks (*p* = 0.003). Similarly, mean prealbumin levels (mg/dL) were 24.91 (SD: 6.92) at study entry and increased to a mean of 26.11 (SD: 6.55) at 24 weeks (*p* = 0.047). These results are shown in Figure 1.

No significant differences were observed in micronutrients or vitamins during the nutritional intervention (12 and 24 weeks) compared with baseline. Exploratory stratified analyses according to IBD subtype (CD vs. UC) did not reveal significant differences in nutritional parameter evolution between groups. These results are summarized in Table 2.

Clinical and biochemical activity (FC and CRP) remained stable during follow-up. Regarding clinical activity, median Harvey–Bradshaw Index and Walmsley Index scores were 0 at baseline as well as at weeks 12 and 24. FC levels showed a downward trend during follow-up, without reaching statistical significance (*p* = 0.067). In contrast, CRP showed statistically significant differences across time points (*p* = 0.013), although mean values remained within low ranges, without clinically relevant changes (Table 2).

Notably, 58.1% of patients (64/110) had low vitamin D levels (<30 ng/mL) at baseline, without a statistically significant improvement in this deficiency during nutritional support follow-up. No association was identified between low vitamin D levels and clinical activity (HBI and Walmsley Index), biochemical activity (FC and CRP), IBD location, or the presence of other immune-mediated inflammatory diseases (IMIDs).

Regarding BMI, 3 patients (2.7%) had BMI < 18.5, 63 patients (57.3%) were normal weight (BMI 18.5–25), and 44 patients (40.0%) were overweight (BMI > 25).

No significant weight changes were observed normal-weight patients after tool use (*p* = 0.20), whereas in overweight patients there was a trend toward weight reduction that did not reach statistical significance (*p* = 0.09). In contrast, patients with obesity achieved a significant weight reduction using both the *t*-test (*p* = 0.00027) and the Wilcoxon test (*p* < 0.0001), with a mean weight loss of −5.7 kg, equivalent to approximately 6% of initial body weight.

No differences were identified in clinical activity (HBI or Walmsley Index) between overweight and normal-weight patients (*p* = 0.75 and *p* = 0.57, respectively). However, a lower mean FC value was observed in overweight patients (166.26 µg/g vs. 445.15 µg/g), although this difference did not reach statistical significance (*p* = 0.18). No differences were observed in CRP levels between the two groups (5.13 mg/L vs. 2.6 mg/L; *p* = 0.09).

No differences were identified in IBD type, disease location, or f biologic therapy line between patients with and without overweight.

Significant improvements were also observed in lifestyle and dietary habits. In particular, there was an increase in physical activity and in the consumption of fruits, vegetables, legumes, and fish (both white and oily). Likewise, a reduction in the consumption of red meat, pastries, and sugar-sweetened beverages was observed, reflecting greater adherence to healthy dietary patterns. These improvements were sustained throughout follow-up, with the largest increases observed in the scores for physical activity (+0.71), daily fruit consumption (+0.81), and vegetable intake (+0.78), as well as a relevant reduction in red meat consumption (−0.88).

Regarding malnutrition risk assessed using the self-administered component of the MUST questionnaire, 17.4% of the sample (13 patients) were at moderate/high risk. No association was found between malnutrition risk according to MUST and clinical activity (HBI, Walmsley Index), biochemical activity (FC, CRP), or IBD type. Notably, most patients with moderate/high risk were of normal weight according to BMI (9 patients, 69.2%), and only 15.3% (2 patients) were underweight (*p* < 0.0001).

When analyzing the evolution of malnutrition risk according to the degree of adherence to the digital nutritional program, patients with a higher percentage of adherence showed greater MUST score improvement at 24 weeks compared with those with lower adherence (*p* = 0.045), while at 12 weeks a nonsignificant trend was observed (*p* = 0.068). Additionally, a significant positive correlation was identified between application use intensity (days of use) and improvement in MUST scores at both 12 weeks (r = 0.26; *p* = 0.022) and 24 weeks (r = 0.25; *p* = 0.034).

In multivariate analysis, digital tool use intensity was independently associated with MUST score improvement at 24 weeks (β = 0.002; *p* = 0.030), after adjusting for age and baseline BMI. Likewise, a higher baseline BMI was associated with greater improvement in MUST (β = 0.045; *p* < 0.001), while age did not show a significant association. The model explained approximately 20% of the observed variability (R^2^ = 0.20).

Quality of life scores assessed using the IBDQ questionnaire were evaluated at three time points: baseline, week 12, and week 24. Median scores were 177.5, 181.5, and 178.0, respectively. No statistically significant differences were observed across time points (χ^2^ = 5.03; *p* = 0.084). The potential impact on the different domains included in the IBDQ (work, social, and mood) was analyzed separately. No significant changes were identified over follow-up in the work domain (median 7 points, *p* = 0.085), social domain (median 7 points, *p* = 0.415), or mood domain (median 6 points, *p* = 0.441).

Before initiation of nutritional support, the median Nootric score was 31 points (IQR: 26–36). After tool use, it increased to 37 points (IQR: 32–43) at 12 weeks and to 40 points (IQR: 36–47) at 24 weeks. These differences reached statistical significance (*p* = 0.018).

Non-adherent patients had significantly lower baseline Nootric score (Mann–Whitney U: *p* = 0.0043), which may suggest lower motivation or a lower perceived relevance of nutrition in disease management. In addition, a moderate positive correlation was observed between the number of days of use and Nootric score improvement (r = 0.45; *p* = 0.000021), indicating that greater adherence to the intervention is associated with greater improvement in the habits and behaviors. A greater improvement in Nootric score was observed in patients using it for more than 83 days at week 12 and more than 125 days at week 24, demonstrating a significant dose–response effect (*p* < 0.001 and *p* = 0.007, respectively)

No significant differences were observed between adherent and non-adherent patients regarding age, sex, baseline disease activity, or baseline fecal calprotectin levels.

Among included patients, frequent access to the tool was identified in 84.2% of users, with use on 47.1% of potential days, totaling 56,175 interactions with recipes (requests for changes, details), 18,660 interactions with the nutritionist regarding diet, and 8047 direct chat messages. Patient satisfaction with the nutritional service was rated at 4.3 out of 5 points.

## 4. Discussion

Our results demonstrate that nutritional support delivered through the mobile digital tool (Nootric^®^) is a feasible and well-accepted strategy, associated with high patient participation and adherence, as well as with a significant improvement in nutritional parameters such as albumin (from 4.38 g/dL to 4.49 g/dL; *p* = 0.003) and prealbumin (from 24.9 mg/dL to 26.1 mg/dL; *p* = 0.047), even in a population without baseline protein–calorie malnutrition. Although albumin and prealbumin values were within normal laboratory ranges at baseline, these findings suggest that the intervention promoted improved protein intake and overall diet quality, which could translate into a potential reduction in subclinical inflammatory status, consistent with recent studies highlighting the interrelationship between nutrition and systemic inflammation in IBD [14,15,16].

The available literature emphasizes that malnutrition is a frequent IBD complication associated with poorer clinical outcomes, higher hospitalization risk, and reduced quality of life [15,16,17,18,19,20]. Although traditionally linked to protein–calorie undernutrition, malnutrition may also coexist with overweight or obesity [16,17]. In our cohort, nearly 40% of patients were overweight, highlighting that malnutrition in IBD should not be understood exclusively as undernutrition, but rather as a broader spectrum of qualitative and quantitative alterations in nutritional status. This finding aligns with Jabłońska et al. [17] and Chicco et al. [18], who also emphasize that diet quality imbalances, even with adequate caloric intake, may worsen the inflammatory course of the disease. These authors propose addressing malnutrition from the perspective of body composition and diet quality rather than focusing solely on body weight. In our series, 17.4% of patients were at malnutrition risk according to the MUST questionnaire, despite most having a normal BMI, underscoring the need for systematic nutritional screening in routine clinical practice.

It is also noteworthy that overweight is a frequent problem in these patients, similar to what is described in Western population, making targeted management of this condition equally necessary. In our cohort, with a 40% prevalence of overweight, we observed a mean weight loss of 6%, which is clinically relevant, as international guidelines recommend weight losses greater than 5% to achieve significant metabolic benefits in obesity [19].

Although Spain is traditionally considered a Mediterranean country, and it is often assumed that both the general population and patients with IBD habitually follow this dietary pattern, our results and those of other recent studies show that this perception does not reflect reality [18,20,21,22]. In fact, patients with IBD frequently adopt restrictive diets, eliminating food groups due to symptom exacerbation fears or lack of adequate nutritional guidance [6,23,24,25]. These practices may contribute to nutritional deficiencies and diet quality imbalances, underscoring the need for enhanced nutritional education and individualized dietary follow-up in this population.

The Mediterranean diet is associated with lower systemic inflammation, a better antioxidant profile, and favorable gut microbiota modulation [4,5,26] PREDIMED study [13,14] results have consolidated its cardiovascular prevention role, and its potential relevance in chronic inflammatory diseases is increasingly recognized. In our experience, the improvement observed in the Nootric score (and the PREDIMED test) after the intervention highlights that healthcare professionals’ perception of adequate adherence to the Mediterranean diet does not always correspond to patients’ actual habits. Our findings reinforce the hypothesis that a nutritional support program, even when delivered digitally, can be an effective complementary tool to improve adherence to this dietary pattern and thereby contribute to the overall well-being of IBD patients.

The use of a digital platform offers additional advantages, including continuous bidirectional communication with patients, content personalization, and flexible follow-up. These characteristics align with ESPEN guideline recommendations, promoting an individualized and multidisciplinary nutritional approach in IBD [8,9]. In our study, the high degree of interaction and patient satisfaction supports the feasibility and acceptance of this model, which could serve as a complement to traditional outpatient visits in units with limited resources. Moreover, digital tools allow for continuous and flexible monitoring, improving adherence to dietary recommendations and supporting the sustainability of results over time.

It is important to clarify that the primary intervention evaluated in this study was the implementation of a structured, dietitian-supervised digital nutritional program delivered through the Nootric^®^ platform. The Mediterranean diet served as the evidence-based dietary framework within this intervention. Therefore, the added value of the study lies not only in promoting Mediterranean dietary adherence but in demonstrating that a digital, supervised strategy can effectively facilitate and sustain these dietary changes in routine clinical practice.

This study has some limitations that should be acknowledged. The absence of a control group limits the ability to establish definitive causality between the digital intervention and the observed improvements. In addition, the relatively low prevalence of severe malnutrition in our cohort may restrict the generalizability of findings to more vulnerable IBD populations. Although we acknowledge that a per-protocol analysis may introduce potential selection bias, given the observational nature of the study and the fact that only patients who used the application were actually exposed to the nutritional intervention, we considered this approach to be the most methodologically appropriate to address our research question. Finally, as a single-center study, external validity may be limited. Nevertheless, the homogeneity of the cohort, the prospective design, and the high level of patient adherence strengthen the reliability and clinical relevance of the findings.

## 5. Conclusions

Digital nutritional intervention using Nootric^®^ proved to be a feasible and well-accepted tool, associated with significant improvements in adherence to the Mediterranean diet and in protein-related nutritional parameters in IBD patients. This support model may represent an effective complementary strategy for comprehensive disease management, particularly in settings with limited access to in-person nutritional follow-up.

## Figures and Tables

**Table 1:** Baseline IBD-related characteristics of the included patients.

Variable	Category	Frequency (n)	Percentage (%)
Mean age (SD)	46.8 años (12.8)		
Sex	Female	58	52.7
	Male	52	47.3
Smoking status	Non-smoker	65	59.1
	Former smoker	20	18.2
	Current smoker	25	22.7
Comorbidities	No	64	58.2
	Yes	46	41.8
Type of IBD	Crohn’s disease	45	40.9
	Ulcerative colitis	65	59.1
BMI	<18.5	53	2.7
	18.5–25	63	57.3
	>25	44	40.0
Other IMIDs	No	84	76.4
	Yes	26	23.6
Type of IMID	Articular	16	61.5
	Cutaneous	8	30.8
	Other	2	7.7
Previous biologic therapies	0	62	56.4
	1	23	20.9
	2	12	10.9
	3	8	7.3
	4	4	3.6
	6	1	0.9
Previous surgeries	0	84	76.4
	1	22	20.0
	2	2	1.8
	5	2	1.8
Flares in the last year	0	88	80.0
	1	16	14.6
	2	5	4.6
	4	1	0.9
Current therapy	Biologics:	88	80.0
	Mesalazine:	14	12.7
	iJAK	2	1.8
	Other	6	5.5

**Table 2:** Evolution of biochemical and nutritional parameters during follow-up.

Variable	Baseline (Mean [SD])	12 Weeks (Mean [SD])	24 Weeks (Mean [SD])	*p* (ANOVA)
Albumin (g/dL)	4.38 (0.26)	4.45 (0.26)	4.49 (0.28)	0.003
Prealbumin (mg/dL)	24.91 (6.92)	24.79 (6.43)	26.11 (6.55)	0.047
Total protein (g/dL)	7.25 (0.43)	7.30 (0.42)	7.31 (0.46)	0.09
Cholesterol (mg/dL)	180.19 (36.29)	178.34 (37.90)	176.34 (35.82)	0.13
Triglycerides (mg/dL)	114.00 (67.36)	109.13 (70.88)	106.13 (56.54)	0.908
Iron (ug/dL)	95.34 (36.95)	91.76 (37.84)	95.53 (34.64)	0.75
Magnesium (mg/dL)	1.90 (0.20)	1.96 (0.19)	1.95 (0.22)	0.26
Vitamin A (ug/mL)	0.58 (0.19)	0.54 (0.17)	0.57 (0.19)	0.12
Vitamin D (ng/mL)	30.58 (14.24)	24.37 (12.54)	26.97 (15.99)	0.06
Vitamin E (ug/mL)	14.49 (4.17)	14.21 (3.89)	13.93 (3.63)	0.33
Vitamin B12 (pg/mL)	414.34 (164.49)	434.25 (179.61)	392.99 (120.51)	0.06
Folic acid (ng/mL)	10.32 (5.36)	9.55 (4.79)	9.22 (4.60)	0.16
Fecal calprotectin (ug/g)	339.8 (876)	25.6 (829.4)	285.9 (869.9)	0.067
C-reactive protein (mg/L)	3.35 (5.95)	3.71 (5.5)	2.92 (4.59)	0.013

## Data Availability

The original contributions presented in this study are included in the article. Further inquiries can be directed to the corresponding author.

## Abbreviations

IBD	Inflammatory bowel disease
CD	Crohn’s disease
UC	Ulcerative colitis
MUST	Malnutrition Universal Screening Tool
HBI	Harvey–Bradshaw Index
SCCAI	Simple Clinical Colitis Activity Index
CRP	C-reactive protein
FC	Fecal calprotectin
BMI	Body mass index
ITT	Intention-to-treat
